# Intertemporal Bargaining in Addiction

**DOI:** 10.3389/fpsyt.2013.00063

**Published:** 2013-08-14

**Authors:** George Ainslie

**Affiliations:** ^1^School of Economics, University of Cape Town, Cape Town, South Africa; ^2^Department of Veterans Affairs, Coatesville, PA, USA

**Keywords:** addiction, hyperbolic discounting of reward, intertemporal choice, self-control, brain imaging of motivation

## Abstract

The debate between disease models of addiction and moral or voluntarist models has been endless, and often echoes the equally endless debate between determinism and free will. I suggest here that part of the problem comes from how we picture the function of motivation in self-control. Quantitative experiments in both humans and non-humans have shown that delayed reward loses its effectiveness in proportion to its delay. The resulting instability of preference is best controlled by a recursive self-prediction process, intertemporal bargaining, which is the likely mechanism of both the strength and the experienced freedom of will. In this model determinism is consistent with more elements of free will than compatibilist philosophers have heretofore proposed, and personal responsibility is an inseparable, functional component of will. Judgments of social responsibility can be described as projections of personal responsibility, but normative responsibility in addiction is elusive. The cited publications that are under the author’s control can be downloaded from www.picoeconomics.org.

Many factors promote the impulse for an addictive activity. In addition to social and informational differences between individuals, the differential attractiveness of such activity is associated with heritable differences in the highs people get from a given activity, differences in their inborn tendency to discount delayed rewards, and adaptation of their brain reward structures to repeated addictive activity (1). Prior factors that have increased the differential reward for addictive activities – even the self-inflicted adaptation factor – could be said to have created a disease, in that the person’s current level of temptation is not subject to voluntary control. Eczema is called a disease, after all, even though the involuntary process is the itching, whereas voluntary scratching is what does the damage. However, discovery of the physical roots of temptation should not obscure the process of motivated choice that is never bypassed in addiction.

Addiction is sometimes identified with physiological patterns such as intoxication, tolerance, and withdrawal, regardless of how the person values them. However, the kind of addiction that a person complains of has two essential elements: temporary preference for inferior rewards, and failure to forestall the recurrent surrender to this preference. The first element arises from the universal over-valuation of imminent rewards, which will produce temporary preferences for them to the extent that the person does not compensate for it. It has been suggested that this over-valuation represents just the arousal of appetites or emotions (2), but a more fundamental pattern is now well documented (3–5). People have inherited a hyperbolic delay discount function from our non-human ancestors:

$$\text{Present value}={\text{Value}}_{\text{0}}/[1+(k\times \text{Delay})]$$

where Value_0_ = value if immediate and *k* is degree of impatience.

This function makes the value of an event inversely proportional to its expected delay, which means that many objectively smaller sooner (SS) rewards will tend to be temporarily preferred to larger later (LL) alternatives when the SS rewards are imminent (Figure 1). The hyperbolic shape of the value/delay function probably survived natural selection because it is a basic psychophysical relation, the same as that for sensory perceptions such as brightness and heaviness (6). In species where instinct generates present reward for future-oriented tasks (hoarding, dam-building, migrating…) this shape is probably not maladaptive, but it gives humans a temptation problem.

**Figure 1:**
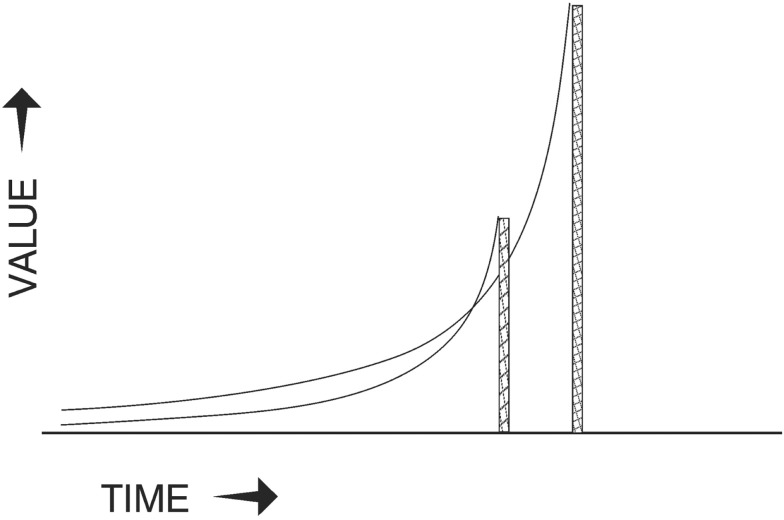
**Hyperbolic curves describing the values of a smaller, sooner (SS) reward and a larger, later (LL) reward as the delay before they will be available decreases**. An early choice (near the left edge) would favor the LL reward, but a choice when the pair is close would favor the SS reward.

Modern people are set up for addictions at birth. Since Isaac Marks’ seminal article (7) many activities have been identified as addictive without the involvement of substances, or even external rewards. Analysis of hyperbolic discounting suggests how the great human ability to coin reward by imagination can be channeled into addictive temptations, by patterns of outcomes that serve as occasions for reward but that make no prediction at all of external rewarding events, as in video games (8). What differentiates the addict from her neighbor is not the strong temptation, when close up, to seek options that she would avoid from a distance. Rather it is the collapse of her resistance to this temptation. This is the other essential element of addiction. We normally reach compromises with our urges, and set boundaries to our bad habits. Understanding this failure – indeed understanding why “compromise” is the right word – will be key to understanding responsibility in addiction.

## Recursive Self-Prediction

Motivation for impulse control comes from the value described by the hyperbolic discount function at long delays, which is proportional to objective value when both alternatives are distant – and much higher for each than is described by either conventional exponential or “quasi-hyperbolic” (cue-driven) functions (9). The need to adopt protections for long term plans is unique to humans, and the number of these that the new human species has been able to learn has been limited: to some extent a person can avoid paths that take her close to SS alternatives, or she can avoid revaluation of them when close, but these strategies will necessarily be hard to maintain for long periods. Alternatively she can set up incentives, especially social ones, that will add deterrence at crucial moments. However, I have argued that a great part of impulse control in an individualistic society is accomplished by the person’s perception that large future benefits depend on regular choices of a particular kind – such as not to drink, spend, or play too much – and that each current choice is a test case for the choices she can expect future selves to make (4, 9). Such self-prediction creates a potentially strong but somewhat rigid mechanism of impulse control, willpower, without the need for a separate organ or faculty. The person can then afford to live closer to temptations, although her self-control will be at risk from her thinking of reasons that a future self should not count a present indulgence as a lapse – that is, from rationalizations.

The person will see a current abstinence as worth the effort if and only if this is necessary to maintain a credible pattern of abstinence, a perception that organizes her relevant choices into a repeated prisoner’s dilemma – or self-enforcing contract – where a current defection jeopardizes future cooperation. The devil is in the word “relevant.” If the person can see how a current consumption should not change her future expectation – “today is special” – then she may get to enjoy it without damaging her long term prospects. Auditioning rationales for their credibility and modifying them accordingly is a recursive, often rapid process. If successful, it maintains the compromise between urges and long term expectations. Rationalizations may not be especially risky when the stakes are low (“I resolve to clean my room today”), but the risk becomes demonstrable when large amounts of incentive hinge on the test, as when a recovering alcoholic decides to try drinking just once. This plan is apt to follow the same logic as the decision of a party to a self-enforcing contract to cheat her partner; defection by a current self often leads to the notorious abstinence violation effect (10; for dieters, see 11). Such a sudden loss of control after a single lapse has sometimes been blamed on a physical stimulation of appetite; however, when alcoholic volunteers are given either alcohol punch or an indistinguishable placebo, the belief that they have had alcohol leads to craving whereas the alcohol itself does not (12).

## Evidence of Recursive Self-Prediction in Will

Recursive (fed-back) processes are notoriously hard to experiment on, even in physical systems (e.g., 13, pp. 191–211), let alone motivational ones, a difficulty which may have concealed their prevalence. Recursive self-prediction is most evident in common intuitions about the motivational consequence of a single lapse in a diet or sobriety, as above, which are sharpened in thought problems such as Kavka’s, Monterosso’s, and Newcomb’s (14). There have been some suggestive experiments: pointing out to subjects that their current choice in a series of SS/LL reward choices may be predictive of their future preferences raises their frequency of LL choices, although not as much as does obligatory commitment for the whole series with their first choice (15, 16). The intertemporal bargaining model fits the properties of will specified by the early psychologists who analyzed will (4, pp. 79–80, 117–120), and predicts the behavior of subjects in 2- and *N*-person prisoner’s dilemma analogs (4, pp. 90–94; 17). However, given that intertemporal bargaining depends on intrapsychic contingencies, we might hope for better evidence from brain imaging.

Unfortunately, imaging experience is rudimentary. Using functional magnetic resonance imaging (fMRI), Luo et al., found that equal preference between a LL reward and a SS alternative cannot be predicted from the activity in brain reward centers when those alternatives are offered singly, outside of the choice situation (18). After establishing a behavioral indifference point, subjects were given chances to respond for each outcome separately. They showed less activity in their reward centers for what had been the LL rewards than for the erstwhile SS rewards, even though they had been indifferent when choosing between these rewards, and were still indifferent when the choice contingency was offered again. That is, the mere establishment of a choice contingency changes the relative value of SS and LL outcomes, in favor of the LL one. The implication is that when impulse control is relevant, motivation beyond what arises from the current rewards themselves must be active. In other studies the lateral frontal cortex has been found to be more active when a subject makes LL choices (e.g., 19), but a recent experiment raises a question of whether this activity is tracking motivation for those LL choices. Luo et al. observed individual subjects’ stochasticity (variability) of SS/LL choice over time (20). By finding the ratio of SS to LL amount that produced equal preference, and then offering differing amounts in the same ratio at the same alternative delays, it was possible in effect to offer a subject the same choice repeatedly in a way that the subject would not recognize, and thus to observe small, spontaneous variations over trials of SS vs. LL preference. Lateral frontal cortical activity was indeed often greater with LL choice, but mostly in subjects who showed relatively great stochasticity – the ones who wavered most. This somewhat counterintuitive finding suggests that activity in lateral frontal cortical “executive centers” may not reflect preference for LL alternatives *per se*, but perhaps represents a response by which subjects compensate for a perceived unreliability of such preference. These studies are far from definitive, but they suggest that self-control in SS/LL choices may be determined by processes on at least two levels above the spontaneous valuation of rewards that is seen in non-choice designs: mental effort, possibly reflected in lateral frontal cortical activity; and a non-effortful evaluative process in which the most stable long term preferences are established – possibly by intertemporal bargaining – which so far lacks fMRI correlates. It may be significant that subjects with relatively great ventromedial prefrontal cortical activity when imagining future selves also prefer LL rewards relatively more than do other subjects (21), but the role of such “prospection” centers in self-control has not been explored.

## Freedom of Will

Assuming that recursive self-prediction is the basis of will, what are the implications for addicts’ responsibility? Intertemporal bargaining increases the power of self-control but also its potential volatility. A recovering alcoholic may notice that she is procrastinating about an unrelated issue and interpret that as evidence of her will being weaker than she thought, which may in turn reduce her expectation of staying sober. That reduction may motivate increased rigidity to prove she is strong, or it may snowball into expectations that are too greatly lowered for their peril to deter a drinking episode. The potential for small or merely symbolic choices to shift much larger motives gives the will an element of chaos, in the technical sense (13). Whatever awareness of this process a person has acquired is apt to weigh on every choice that she notices to be evidence of her predilections. This extra motivation provides a rationale for strength of will – and also, arguably, for a mechanism that meets common definitions of freedom of will.

As characterized by Richard Holton, people’s insistence on the freedom of will largely stems from two kinds of experience: the unpredictability of one’s own behavior (“For all I know, I might have done otherwise”) and the initiation of one’s behavior (“Action is experienced as something that the agent instigates, rather than something that just happens to the agent as the result of the state that they were antecedently in” – 22). We do not feel as if we are passively responding to the incentives we detect. Chaotic systems such as weather have been proposed as models for the unpredictability of our choices (23), but critics have pointed out that being buffeted by the weather would not feel like being free, more like having seizures (24, p. 231). However, the feeding back of tentative choices to the process of choice itself makes the agent a participant in her motivational weather. In a simple linear choice I might decide how much food to take at a buffet lunch on the basis of how hungry I am or how long I expect to go without food in the afternoon. But if I am a restricted eater those considerations may be overshadowed by estimating whether the food I take looks like I am lapsing, and knowing that the prospect of lapsing may increase my hunger. [Recursive self-prediction as a cause of “conditioned” (cue-driven) appetite is discussed in 5, pp. 222–229]. The latter, recursive estimation is of the same cognitive kind as the linear, but the sensation that my making estimates is changing those estimates as I make them has an added quality. Choosing under the influence of the choice itself pulls attention back from the ostensible alternatives to an inward dialog, one that should produce a feeling of agency at the same time that it makes choice unpredictable from its original incentives. The outcome is still strictly determined, of course, but recursively rather than linearly, a distinction that keeps the self from being that old bugbear of determinism, a throughput – a mere conveyor of incentives (fuller discussion in 25).

## Responsibility for Addictive Choices

Much of the effort that has gone into finding rationales for free will stems from its supposed necessity for holding people responsible for their choices (26). A theory that derives the functional properties of free will from recursive self-prediction is still deterministic, and thus does not seem at first to be a solution. However, the way that this theory relates strength to freedom of will suggests a functional framework for responsibility without indeterminacy – and thus a space in which the voluntarist model of addiction can be valid. Rather than testing this possibility by the truth or falsehood of determinism, it makes more sense for us to look at the practical roots of responsibility. Psychology has generally assumed that a personal sense of responsibility comes from internalization of pressure that is brought to bear by parents and other socializers, but with the logic of intertemporal bargaining their precepts cease to be needed to provide incentive, only to suggest compromises between internal interests. A serious lapse threatens such compromises, not because the person makes a decision to punish herself but because of the realistic fall in her expectation of future self-control. (This model is not contradicted by social psychology experiments in which subjects who read statements espousing determinism subsequently indulge in minor antisocial behaviors – see 22.)

Personal responsibility is thus an operational component of will. Its inseparability from volition raises the possibility that when people judge someone else to be guilty, they are projecting their own experience with lapses – feeling personal guilt empathically: “If I were in her shoes, I would feel guilty.” That is, our understanding of other people’s responsibility comes from our intuition of our own. Such a process would supply the element of deservingness that is missing from determinist models that explain social responsibility as a manipulation to create deterrence, which are intuitively unsatisfying (27). Thus for both personal and social responsibility, the truth of determinism is not relevant (discussed in 25). However, the question remains of how responsibility is affected by addiction.

An addict undeniably faces disease factors, but my argument is that they operate *through* motivation, not instead of it. Addiction becomes “hopeless” when the addict has no rationale by which abstention at crucial choice points would sufficiently increase the believability of her future sobriety. As described above, such belief is sensitively dependent on many self-generated signs and symbols, but at some point the addict has tried and spoiled all the ones she can think of. She is not sick in the sense of being beyond motivation, but her inability to propose any credible intertemporal deal constitutes a kind of bankruptcy, and she could be argued thus not to be responsible – except that, unlike a financial bankrupt, she sometimes has a sudden epiphany that re-orders her accounts (28). The advice of the Anonymous organizations that the addict is helpless against her addiction does not imply the irrelevance of intertemporal bargaining, but rather the danger of trying rationalizations where the bargaining is full of mistrust. Their injunction to resolve sobriety for only 1 day at a time aims at restarting the bargaining process with a low level of trust. To acknowledge helplessness against a temptation without attempting to renounce it forever sounds illogical, but it is often the only successful compromise after long histories of grand resolution and total collapse. These organizations have intuited most of the tactics that an intertemporal bargaining model would prescribe (which is not to argue for or against their clinical effectiveness).

## Conclusion

As is the case with so many ancient debates, both the disease theory and the moral theory of addiction have part of the truth on their side. The physical roots of addictive temptation are increasingly known, as is their unequal distribution among individuals and within the same individual at different points in her addictive history. In her current moment forces gathered by heredity, exposure, and even her own past behavior are givens, and could fairly be judged to constitute a disease. However, their force is still one of temptation, even when giving in promises only a joyless oblivion instead of the pain of short-lived self-control. The error of policy-makers who rely on negative incentives is not their belief that addictive behavior is motivated, but their miscalculation of how much punishment can make up for the bankruptcy of a person’s intertemporal bargaining process. Unless they have experienced such bankruptcy themselves, trying to put themselves in the addict’s shoes leads them to conclude that she just needs an extra push, whereas in fact she needs to re-establish a relationship with her prospective future selves. Certainly cures for temptation itself can be a factor – for instance, buprenorphine for opiate craving – as can simply structuring modest incentives with a view toward immediacy and reliability (e.g., 29). And certainly, blanket forgiveness of bad deeds that have sprung from an addiction would create perverse incentives, “moral hazard.” But beyond these straightforward contingencies, the difference between sobriety and ruin often lies with the turnings of intertemporal bargaining, which defy simplification.

## Conflict of Interest Statement

The authors declare that the research was conducted in the absence of any commercial or financial relationships that could be construed as a potential conflict of interest.
